# Anesthesia Modality in Intracranial Stenting for Acute Stroke—A Sub-Analysis of the RESISTANT International Registry

**DOI:** 10.1007/s00062-026-01619-7

**Published:** 2026-02-03

**Authors:** João André Sousa, Marta Olivé-Gadea, Francesco Diana, Johannes Kaesmacher, Adnan Mujanovic, Serdar Geyik, Songul Senadim, Amedeo Cervo, Andrea Salcuni, Mariangela Piano, Manuel Moreu, Alfonso López-Frías, Ameer Hassan, Samantha Miller, Elena Zapata-Arriaza, Asier de Albóniga-Chindurza, Mauro Bergui, Stefano Molinaro, Fabio Gomes, Joao Sargento-Freitas, Andrea Alexandre, Alessandro Pedicelli, Jeremy Hofmeister, Paolo Machi, Luca Scarcia, Erwah Kalsoum, Jose Amorim, Torcato Meira, Santiago Ortega Gutierrez, Aaron Rodriguez-Calienes, Leonardo Renieri, Francesco Capasso, Daniele Romano, Eduardo Bárcena-Ruiz, David Seoane, Mohamad Abdalkader, Piers Klein, Thanh N. Nguyen, Catarina Perry, Isabel Fragata, Dileep Yavagal, Jude Charles, Jose Rodriguez Castro, Pedro Vega, Atilla Özcan Özdemir, Zehra Uysal Kocabaş, Stanislas Smajda, Sadiq Al Salman, Jane Khalife, Tudor Jovin, Francesco Biraschi, Francesca Ricchetti, Pedro Castro, Luis Albuquerque, Adnan Siddiqui, Vinay Jaikumar, Pedro Navia, Nikos Ntoulias, Marios Psychogios, Mariano Velo, Joaquin Zamarro, Gonzalo De Paco, Yazan Ashouri, Mohammad AlMajali, Juan F. Arenillas, Alicia Sierra, Michele Romoli, João Pedro Marto, Shadi Yaghi, Marc Ribo, Alejandro Tomasello, Manuel Requena

**Affiliations:** 1 Stroke Unit, Hospitais da Universidade de Coimbra, Coimbra, Portugal; 2 Faculdade de Medicina, University of Coimbra, Coimbra, Portugal; 3 Vall d’Hebron Hospital Universitari, Barcelona, Spain; 4 Link Campus University, Rome, Italy; 5 University Hospital of Bern, Bern, Switzerland; 6 Centre Hospitalier Universitaire de Tours, Tours, France; 7 Istanbul Aydın University, Istanbul, Turkey; 8 Ospedale Niguarda Ca’ Granda, Milan, Italy; 9 Hospital Clínico San Carlos, Madrid, Spain; 10 Valley Baptist Medical Center, Harlingen, United States; 11 Hospital Universitario Virgen del Rocío, Seville, Spain; 12 Institute of Biomedicine of Seville, Seville, Spain; 13 Azienda Ospedaliera Citta’ della Salute e della Scienza di Torino, Turin, Italy; 14 Agostino Gemelli University Polyclinic, Rome, Italy; 15 Fondazione Policlinico Universitario A. Gemelli, Agostino Gemelli University Polyclinic, Rome, Italy; 16 University Hospital of Geneva, Geneva, Switzerland; 17 Hôpitaux Universitaires Henri-Mondor, Créteil, France; 18 Hospital Braga, Braga, Portugal; 19 University of Iowa, Iowa City, United States; 20 Azienda Ospedaliero-Universitaria Careggi, Florence, Italy; 21 Ospedali Riuniti San Giovanni di Dio e Ruggi d’Aragona, Salerno, Italy; 22 Hospital Universitario 12 de Octubre, Madrid, Spain; 23 Boston Medical Center, Boston, United States; 24 Centro Hospitalar de Lisboa Central, Lisbon, Portugal; 25 University of Miami, Coral Gables, United States; 26 Central University Hospital of Asturias, Oviedo, Spain; 27 Interventional Neuroradiology Chair of the University of Oviedo (CENIT), University of Oviedo, Oviedo, Spain; 28 Eskişehir Osmangazi University, Eskişehir, Turkey; 29 Hôpital Rothschild, Paris, France; 30 Cooper University Health Care, Camden, United States; 31 Policlinico Umberto I, Rome, Italy; 32 Hospital de São João, Porto, Portugal; 33 University at Buffalo, State University of New York, Buffalo, United States; 34 Hospital Universitario La Paz, Madrid, Spain; 35 University Hospital of Basel, Basel, Switzerland; 36 Neuroradiology Unit, Department of Biomedical, Dental Science and Morphological and Functional Images,, University of Messina, Messina, Italy; 37 Hospital Universitario Virgen de la Arrixaca, Murcia, Spain; 38 Toledo Hospital, Toledo, United States; 39 Hospital Clínico Universitario de Valladolid, Valladolid, Spain; 40 Ospedale “M. Bufalini” di Cesena, Cesena, Italy; 41 Hospital de Egas Moniz, Lisbon, Portugal; 42 Brown University, Providence, United States

**Keywords:** Ischemic stroke, Endovascular treatment, Stents, Anesthesia

## Abstract

**Purpose:**

The optimal anesthetic approach for intracranial stenting in acute stroke remains unclear. We compared outcomes of patients under general anesthesia (GA) versus local anesthesia or conscious sedation.

**Methods:**

The RESISTANT registry is a multicenter observational study on acute intracranial stenting during thrombectomy. Patients treated between January 2016 and June 2023 were included and stratified into GA and local anestesia/conscious sedation groups. The primary outcome was an adjusted shift analysis of the modified Rankin Scale (mRS) at 90 days. Secondary outcomes included mRS 0–2 at 90 days and final modified Thrombolysis in Cerebral Infarction (mTICI) 2c/3 scores. Safety outcomes were symptomatic intracranial hemorrhage (sICH) and mortality. Adjusted ordinal and logistic regression with mixed-effects models were performed.

**Results:**

Of 876 patients, 445 (50.8%) received GA. Median age was 67 years [59–77]; 567 (64.8%) were men. No differences were found in 90-day mRS (adjusted common OR = 1.256 [0.887–1.780], *p* = 0.199). Rates of functional independence (39.0% vs 44.5%; aOR = 0.956 [0.606–1.507], *p* = 0.846), mTICI 2c/3 (68.9% vs 68.7%; aOR = 0.941 [0.602–1.471], *p* = 0.790), and sICH (8.0% vs 8.6%; aOR = 0.769 [0.374–1.584], *p* = 0.477) were comparable. In-hospital (23.0% vs 12.0%; aOR = 2.39 [1.35–4.22], *p* = 0.003) and 90-day mortality (33.3% vs 21.1%; aOR = 2.017 [1.227–3.315], *p* = 0.006) were higher in the GA group.

**Conclusion:**

In patients undergoing intracranial stenting during thrombectomy, anesthesia modality was not associated with better outcomes. GA was linked to higher mortality, likely due to indication bias.

**Supplementary Information:**

The online version of this article (https://doi.org/10.1007/s00062-026-01619-7) contains supplementary material, which is available to authorized users.

## Introduction

Patients who fail to recanalize during endovascular stroke treatment have poor outcomes [1, 2], potentially even worse than those who undergo medical management [2]. Observational data suggest that acute intracranial stenting, whether used as a bailout rescue technique or for residual flow-limiting stenosis may be associated with improved clinical outcomes [3, 4].

Randomized clinical trials on non-acute intracranial stenting for severe atherosclerotic disease management, such as SAMMPRIS [5], WEAVE [6] and CASSIS [7] have protocolized general anesthesia (GA) to prevent patient agitation and procedural complications. Additionally, in the acute setting of intracranial thrombectomy, GA has been associated with higher recanalization rates [8, 9] and better outcomes at 3 months [10]. However, GA may delay reperfusion, a key prognostic factor, particularly in settings where it is not routinely used [11, 12]. It may also lead to deleterious hemodynamic changes, such as hypotension [9] which can impair collateral circulation [13], which is particularly important in intracranial atherosclerotic disease—a major contributor to failed recanalization and the subsequent need for bailout stenting.

Current guidelines recommend tailoring the anesthesia strategy during endovascular stroke treatment based on individual patient risk factors and procedural considerations [14]. Therefore, we aim to provide insight into the impact of anesthesia strategy in patients who underwent acute intracranial stenting during endovascular stroke treatment, using data from a large multicenter international registry. We compare the efficacy and safety outcomes of patients who underwent GA versus those who received local anesthesia, monitored anesthesia care and/or conscious sedation.

## Methods

### Study Design

We conducted a pre-planned secondary analysis of the RESISTANT registry. This international 36-center observational retrospective study included adult patients that underwent intracranial stenting during endovascular treatment of an acute ischemic stroke from January 2016 to June 2023. Local site investigators, who were unblinded to the intervention, reported the variables and outcomes. This substudy was conducted according to STROBE guidelines.

### Ethical Considerations and Data Statement

The RESISTANT study protocol was approved by the coordinating center Institutional Review Board which waived the requirement for patient consent due to the retrospective nature of the study. Aggregate data that supports this study can be shared upon reasonable request to the RESISTANT principal investigator.

### Study Groups

Patients from the retrospective RESISTANT cohort were included in this substudy and divided into two groups based on the anesthesia modality chosen by the treating physician at the time of stenting. We performed an as-treated analysis.

### Study Variables

The baseline variables collected in the study included demographic factors (age, sex); pre-morbid modified Rankin Scale (mRS); vascular risk factors (hypertension, dyslipidemia, diabetes, atrial fibrillation, prior transient ischemic attack/stroke, coronary artery disease, smoking status); usual antithrombotic use; and the presence of known intracranial atherosclerotic disease. Additionally, imaging and stroke severity measures were recorded, including Alberta Stroke Program Early CT Score (ASPECTS), National Institutes of Health Stroke Scale (NIHSS) on admission, intravenous thrombolysis use, and occlusion location. Procedural details were also collected, such as stenting strategy (front-line vs. rescue), stent type (balloon-expandable vs. self-expandable), thrombectomy strategy (stent retriever, aspiration, or combined technique), number of passes before stenting, and modified Treatment in Cerebral Ischemia (mTICI) score before stenting. Furthermore, periprocedural antithrombotic use and time from onset to recanalization were also recorded.

### Study Outcomes

The primary outcome was mRS at 90 days (shift analysis). Secondary outcomes included functional independence (mRS 0–2 at 90 days), complete to near to complete recanalization (mTICI 2c/3), early neurological improvement (ENI) defined as NIHSS decrease ≥ 4 at 24–48 h, change in NIHSS (difference between NIHSS at 5 days or discharge and baseline), symptomatic intracranial hemorrhage (sICH) defined as intracranial hemorrhage causing new or worsening neurologic symptoms within 24 h following endovascular treatment (EVT), parenchymal hemorrhage (PH1 or PH 2) according to European Cooperative Acute Stroke Study [15] and mortality during admission and at 90 days.

### Statistical Plan

Descriptive statistics were calculated for all study variables. Continuous variables were summarized as medians with interquartile ranges and compared using the Wilcoxon rank-sum test for two-group comparisons. For comparisons involving more than two groups, the Kruskal-Wallis test was used for non-normally distributed data, while ANOVA was applied when the assumption of normality was met. Categorical variables were reported as counts with frequencies and compared using the Chi-squared test or Fisher’s exact test, as appropriate.

For the primary outcome, we used a mixed-effects ordinal regression model (Cumulative Link Mixed Model with Laplace Approximation) to assess the likelihood of a worse shift in mRS, with categories 5 and 6 pooled together, while accounting for hospital-level clustering. For binary secondary outcomes (mRS 0–2, ENI, TICI 2c/3, sICH, PH, and 90-day mortality), we applied mixed-effects logistic regression using a Generalized Linear Mixed Model with a binomial distribution and logit link function, incorporating hospital-level clustering as a random effect. For continuous secondary outcomes (i. e, change in NIHSS), we used a linear mixed-effects model with hospital-level clustering as a random effect. All models were adjusted for age, sex, baseline NIHSS, pre-stroke mRS, ASPECTS, occlusion location (proximal vs. distal), anterior vs. posterior circulation, tandem lesion, intravenous thrombolysis and number of passes previous to stent deployment. We reported common odds ratios (cOR) for ordinal outcomes, odds ratios (OR) for binary outcomes, and regression coefficients (β) for continuous outcomes, each with 95% confidence intervals (CIs). A *p*-value of  0.05 was considered the threshold for statistical significance, without adjustments for multiple comparisons. Missing data were addressed through pairwise deletion, and analyses were conducted using an available-case approach. All statistical analyses were performed using R software (version 4.3.0, R Foundation for Statistical Computing, Vienna, Austria).

## Results

### Baseline Characteristics

Among the 876 patients in the RESISTANT registry, 445 (50.8%) underwent GA. The median [IQR] age was 67 [59–77] years, and the majority were men (567, 64.8%). Table 1 summarizes baseline characteristics and demographics based on anesthetic modality. Patients in the GA group had a higher proportion of posterior circulation strokes (37.6% vs 18.9%, *p*  0.001). The distribution of the anesthetic strategy stratified by each respective stroke center can be found in supplementary table 1.

**Table 1:** Baseline characteristics according to anesthesia modality.

Variable	Number of patients with data available	All patients (*n* = 876)	General Anesthesia (*n* = 445, %)	Conscious sedation/Local Anesthesia (*n* = 431)	*P*-value
*Age (median, IQR)*	876	67.0 (59.0, 77.0)	68.0 (59.0, 77.0)	67.0 (59.0, 77.0)	0.511
*Female Sex (n,%)*	875	308 (35.2%)	149 (33.5%)	159 (37.0%)	0.279
*Prestroke mRS 0–2*	743	683 (91.9%)	331 (90.2%)	352 (93.6%)	0.087
*Prestroke mRS (median, IQR)*	743	0 (0–1)	0 (0–1)	0 (0–1)	0.006
Anterior circulation	525	0 (0–1)	0 (0–2)	0 (0–1)	0.002
Posterior circulation	215	0 (0–1)	0 (0–1)	0 (0–1)	0.899
*Hypertension*	869	641 (73.8%)	333 (75.0%)	308 (72.5%)	0.397
*Hyperlipidemia*	868	406 (46.8%)	214 (48.3%)	192 (45.2%)	0.355
*Diabetes Mellitus*	867	317 (36.6%)	153 (34.5%)	164 (38.7%)	0.206
*Atrial Fibrillation*	867	121 (14.0%)	61 (13.8%)	60 (14.2%)	0.871
*TIA/Stroke History*	867	242 (27.9%)	117 (26.4%)	125 (29.5%)	0.314
*Coronary Artery Disease*	866	115 (13.3%)	63 (14.2%)	52 (12.3%)	0.403
*Smoker (History or Current)*	862	360 (42.0%)	173 (39.3%)	187 (44.7%)	0.108
*Previous Anticoagulation Use*	864	133 (15.4%)	65 (14.7%)	68 (16.1%)	0.586
*Previous Antiaggregation Use*	864	261 (30.2%)	136 (30.9%)	125 (29.5%)	0.648
*Known ICAD*	865	77 (8.9%)	38 (8.6%)	39 (9.2%)	0.749
*ASPECT Score*	836	9.0 (8.0, 10.0)	9.0 (8.0, 10.0)	9.0 (8.0, 10.0)	**0.001**
*NIHSS Admission*	853	12.0 (7.0, 19.0)	13.0 (8.0, 20.0)	12.0 (7.0, 18.0)	0.113
*IVT*	875	202 (23.1%)	109 (24.5%)	93 (21.6%)	0.314
*Occlusion location*					
Anterior circulation	872	624 (71.6%)	277 (62.4%)	347 (81.1%)	**0.001**
Posterior circulation		248 (28.4%)	167 (37.6%)	81 (18.9%)	
*Occlusion location*					
Proximal (ICA‑T, M1, Basilar, Vertebral)	872	772 (88.5%)	400 (90.1%)	372 (86.9%)	0.141
Distal (M2, M3, M4, ACA, PCA)		100 (11.5%)	44 (9.9%)	56 (13.1%)	
*Tandem Lesion*	875	43 (4.9%)	17 (3.8%)	26 (6.0%)	0.128
*Stenting strategy*					
Front-line	875	116 (13.3%)	62 (14.0%)	54 (12.5%)	0.531
Rescue-stenting		759 (86.7%)	382 (86.0%)	377 (87.5%)	
*Stent type*					
Balloon-expandable	873	176 (20.2%)	87 (19.6%)	89 (20.8%)	0.647
Self-expandable		697 (79.8%)	358 (80.4%)	339 (79.2%)	
*Thrombectomy Strategy*					
Stent Retriever Only	739	121 (16.4%)	71 (18.9%)	50 (13.8%)	0.097
Aspiration Only		168 (22.7%)	77 (20.5%)	91 (25.1%)	
Combined Technique		450 (60.9%)	228 (60.6%)	222 (61.2%)	
*Number of passes pre-stenting*	873	2.0 (1.0, 3.0)	2.0 (1.0, 3.0)	2.0 (1.0, 3.0)	0.389
*TICI pre-stenting*					
TICI 0–2a	789	469 (59.4%)	249 (62.6%)	220 (56.3%)	0.072
TICI 2b–3		320 (40.6%)	149 (37.4%)	171 (43.7%)	
*Periprocedural antiplatelet*					
None	876	49 (5.6%)	22 (4.9%)	27 (6.3%)	0.255
Oral antiplatelet therapy		185 (21.1%)	105 (23.6%)	80 (18.6%)	
IV P2Y12		92 (10.5%)	43 (9.7%)	49 (11.4%)	
GPI		550 (62.8%)	275 (61.8%)	275 (63.8%)	
*Procedural heparin*					
*No*	760	580 (76.3%)	292 (74.9%)	288 (77.8%)	0.630
*Lines/saline*		35 (4.6%)	19 (4.9%)	16 (4.3%)	
*IV*		145 (19.1%)	79 (20.3%)	66 (17.8%)	
*Onset-Recanalization (min)*	717	390.0 (254.0, 655.5)	377.5 (244.5, 644.8)	408.0 (266.0, 662.5)	0.191

### Primary and Secondary Outcomes

**Fig. 1:**
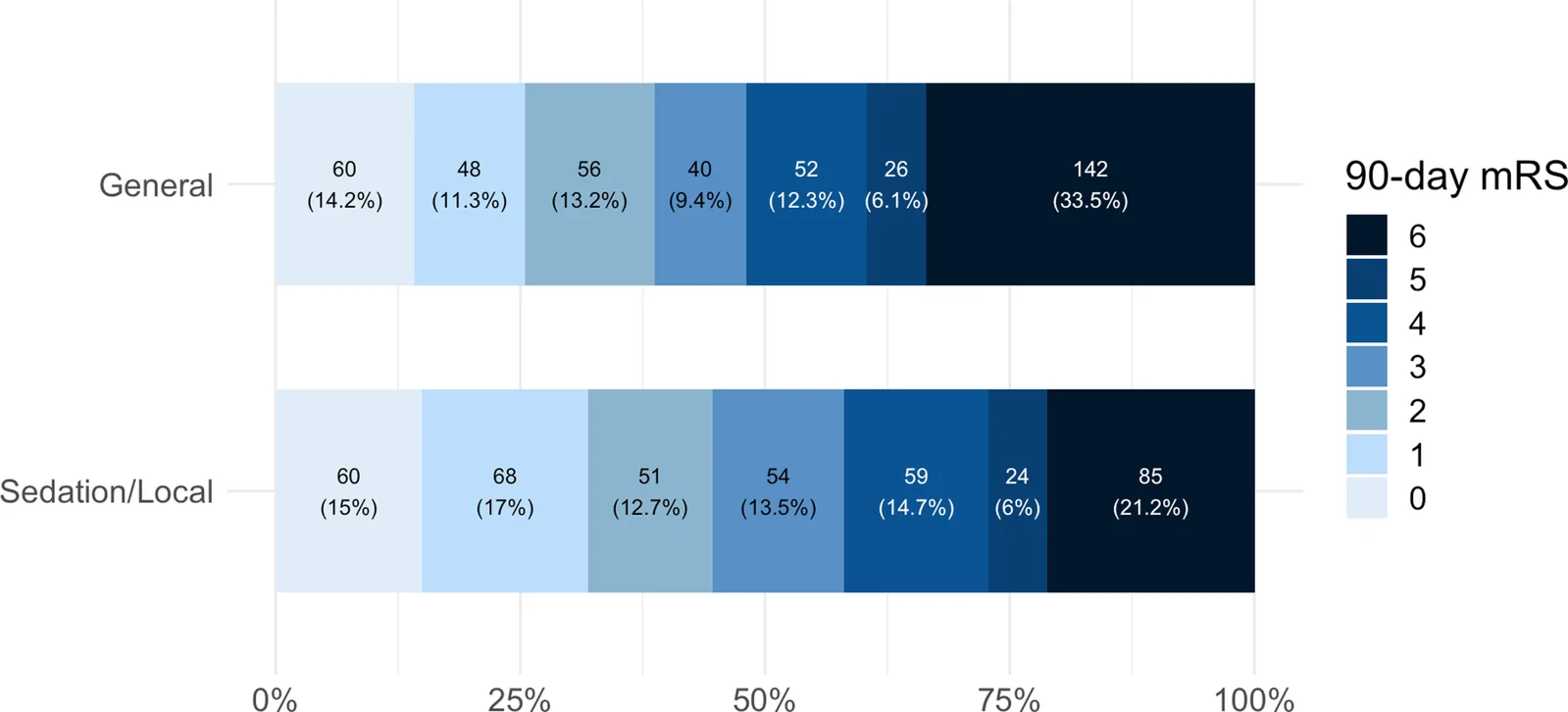
Distribution of 90-Day Modified Rankin Scale Scores by Anesthesia Modality

In an unadjusted analysis, a shift towards a worse mRS at 90 days was observed in patients who underwent GA (common OR = 1.78 [1.32–2.4], *p*  0.001). However, after adjusting for covariates, GA was not statistically associated with worse mRS (adjusted cOR = 1.256 [0.887–1.780], *p* = 0.199). Figure 1 shows the 90-day mRS distributions in GA and local anesthesia/conscious sedation groups.

A similar number of passes was performed prior to stent deployment in each anesthesia modality subgroup (2 [1–3] vs. 2 [1–3], *p* = 0.389), and a comparable final mTICI 2c/3 rate was achieved after stenting (68.9% vs. 68.7%; aOR = 0.941 [0.602–1.471], *p* = 0.790). During hospital admission, the anesthesia modality was not associated with the rate of early neurological improvement (39.6% vs. 35.9%; aOR = 1.114 [0.744–1.667], *p* = 0.601) or change in NIHSS score (median 1.0 [−3.0 to 7.0] vs. 1.0 [−3.0 to 6.0]; aβ = −0.289 [−1.94 to 1.36], *p* = 0.730). At 90 days, GA was not associated with higher odds of functional independence (aOR = 0.956 [0.606–1.507], *p* = 0.846). Table 2 shows the association between GA and secondary outcomes.

**Table 2:** Association between general anesthesia and outcomes in patients who underwent intracranial stenting during endovascular stroke treatment.

	General anesthesia	No-general anesthesia	*P* value	Main analysis			
				Unadj (95% CI)	*P* value	Adj. (95% CI)	*P* value
90-day mRS 0–2	166/426 (39.0%)	179/402 (44.5%)	0.105	*N* = 828, OR = 0.639 (0.450, 0.906)	0.012	*N* = 662,aOR = 0.956 (0.606, 1.507)	0.846
TICI 2c/3	303/440 (68.9%)	294/428 (68.7%)	0.956	*N* = 868, OR = 1.154 (0.804, 1.656)	0.438	*N* = 686,aOR = 0.941 (0.602, 1.471)	0.790
Early Neurological Improvement	161/407 (39.6%)	146/407 (35.9%)	0.278	*N* = 814, OR = 1.167 (0.822, 1.657)	0.388	*N* = 663,aOR = 1.114 (0.744, 1.667)	0.601
Change in NIHSS (∆)	1.0 (−3.0, 7.0)	1.0 (−3.0, 6.0)	0.830	*N* = 814,β = 0.044(−1.5, 1.4)	0.953	*N* = 663, aβ = −0.289 (−1.94, 1.36)	0.730
sICH	34/436 (7.7%)	36/417 (8.6%)	0.635	*N* = 853, OR = 0.997(0.580–1.714)	0.993	*N* = 682,aOR 0.765(0.371–1.575)	0.467
Parenchymal Hemorrhage	20/415 (4.8%)	25/394 (6.6%)	0.331	*N* = 809, OR = 0.842(0.423–1.676)	0.625	*N* = 657,aOR = 0.752(0.343–1.649)	0.478
In-hospital mortality	101/439 (23%)	50/415 (12%)	**0.001**	*N* = 854, OR = 3.54 (2.25–5.60)	>**0.001**	*N* = 686,aOR = 2.39 (1.35–4.22)	**0.003**
Mortality at 90 days	142/426 (33.3%)	85/402 (21.1%)	**0.001**	*N* = 828, OR = 2.755 (1.851, 4.099)	**0.001**	*N* = 662,aOR = 2.017 (1.227, 3.315)	**0.006**

Odds ratios and β coefficients with 95% confidence intervals were calculated using logistic and linear mixed-effects regression models, both unadjusted and adjusted for age, sex, baseline NIHSS, pre-stroke mRS, ASPECTS, proximal vs. distal vessel occlusion, anterior vs. posterior circulation, tandem lesion, intravenous thrombolysis, and site/cluster

### Safety Outcomes

Regarding the procedure, the overall rate of complications was similar between groups (Table 3). The incidence of contrast extravasation and/or vessel perforation was higher in the local anesthesia/conscious sedation group (32/427, 7.5%) compared to the GA group (16/441, 3.6%) (*p* = 0.013). The rate of parenchymal hemorrhage was similar between groups (4.8% vs. 6.6%, aOR = 0.752 [0.343–1.649], *p* = 0.478). The incidence of sICH was also comparable (7.7% vs. 8.6%, aOR = 0.765 [0.371–1.575], *p* = 0.467). Mortality during admission (23.0% vs 12.0%; aOR = 2.39 [1.35–4.22], *p* = 0.003) and at 90 days was significantly higher in the GA group (33.3% vs. 21.1%, aOR = 2.017 [1.227–3.315], *p* = 0.006).

**Table 3:** Rates of procedural complications stratified by anesthesia modality.

	GA (*n* = 441) (%)	No GA (*n* = 427) (%)	*P*-value
No complication	393 (89.1)	361 (84.5)	0.119
Intraprocedural contrast extravasation/vessel perforation	16 (3.6)	32 (7.5)	
Dissection	11 (2.5)	11 (2.6)	
Femoral/retroperitoneal hematoma	6 (1.4)	4 (0.9)	
Other	15 (3.4)	19 (4.4)	

### Subgroup Analysis

**Fig. 2:**
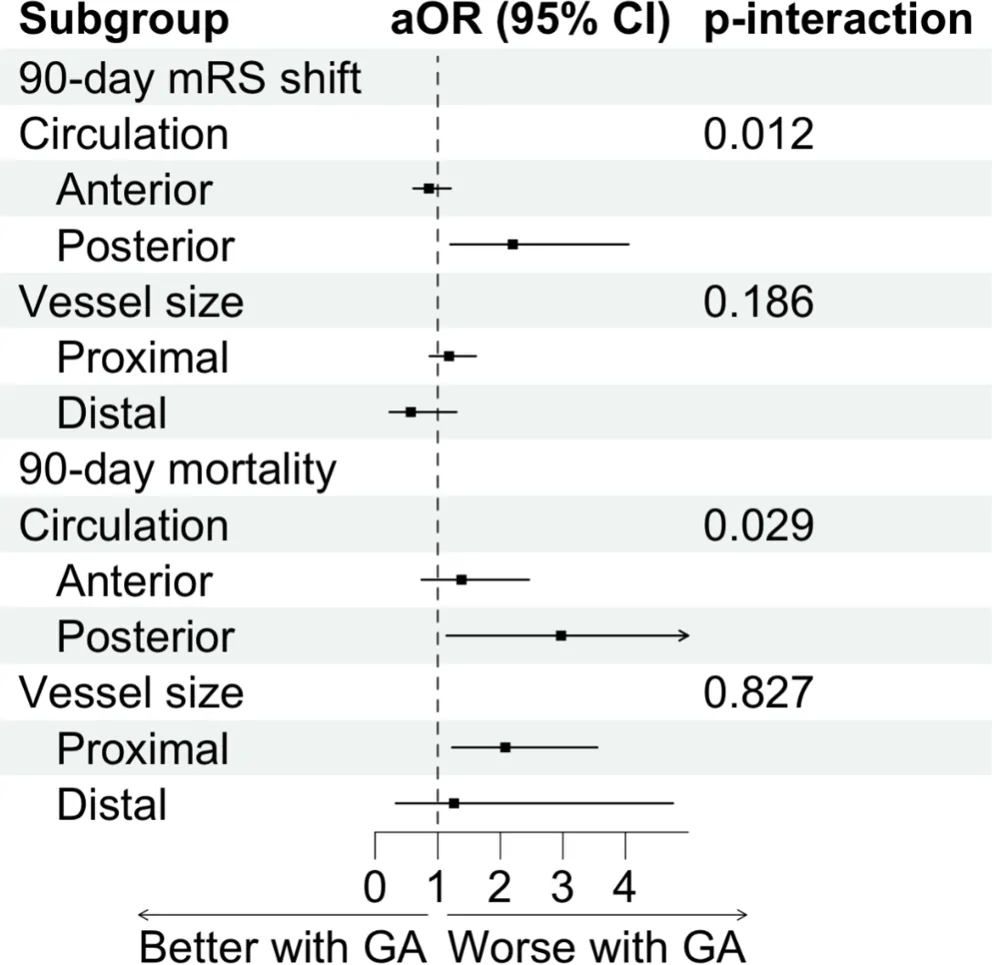
Subgroup Analysis of 90-Day Modified Rankin Scale Shift and Mortality

As shown in Fig. 2, a significant interaction by circulation location and mRS 90-day shift was observed (*p*-interaction = 0.012). In patients with posterior circulation stroke, GA was significantly associated with worse mRS at 90 days (aOR = 2.20, 95% CI: 1.20–4.05), whereas this association was not observed in anterior circulation strokes (aOR = 0.86, 95% CI: 0.61–1.21). Supplementary figure 1 shows the distribution of 90-day mRS stratified by anterior and posterior circulation strokes. No significant interaction was found between GA and mRS shift based on vessel size (*p*-interaction = 0.186), with similar estimated effects in proximal occlusions (aOR = 1.18, 95% CI: 0.87–1.61) and distal occlusions (aOR = 0.57, 95% CI: 0.23–1.30). For 90-day mortality, GA was associated with higher odds of death in posterior circulation strokes (aOR = 2.97, 95% CI: 1.14–7.78), but not in anterior strokes (aOR = 1.38, 95% CI: 0.74–2.46), with a significant interaction by circulation location (*p*-interaction = 0.029). No significant interaction was found based on vessel size (*p*-interaction = 0.827).

## Discussion

This study found that performing acute intracranial stenting under GA during endovascular stroke treatment is not associated with improved technical success, efficacy or safety outcomes compared to other anesthesia modalities. In contrast, we observed a higher 90-day mortality in the GA group. To the best of our knowledge, this represents the largest analysis to date examining the impact of anesthesia modality in this specific endovascular setting.

Taken together, these findings suggest that changing the anesthetic strategy in anticipation of, or in response to, the need for intracranial stent deployment during an EVT—specifically converting from local anesthesia or conscious sedation to GA—does not appear to confer any additional technical or clinical advantage.

A similar retrospective study of patients with anterior circulation large vessel occlusion stroke who underwent rescue intracranial stenting or angioplasty compared 78 patients treated with GA vs. 78 without GA. The study found that both strategies performed comparably in terms of 90-day mRS, successful reperfusion, sICH, procedural complications, and 90-day mortality. Other previous smaller studies have suggested the feasibility and safety of preforming intracranial stenting without GA. Our findings are largely consistent, except for an increased 90-day mortality observed in the GA cohort.

This difference may be explained by the inclusion of both anterior and posterior circulation strokes in our cohort, with posterior circulation strokes being overrepresented in the GA group and subgroup analysis indicating an association between GA and mortality in posterior, but not anterior, circulation strokes. Even after adjusting our models for stroke localization and severity, this finding could reflect an indication bias where GA is often preferred in more severe cases—particularly in patients with decreased consciousness or compromised airway protection or cases in which deterioration occurred and a conversion from local anesthesia/conscious sedation to GA was required. However, although selection bias likely plays a role, we should also acknowledge that GA and prolonged mechanical ventilation may possibly contribute to worse outcomes due to an increased risk of complications such as pneumonia. Patients with posterior circulation strokes and a presumed unfavorable prognosis may be kept intubated, leading to a potential self-fulfilling prophecy. In fact, a meta-analysis of observational studies that included a total of 1351 patients has shown that in posterior circulation strokes, GA is associated with higher mortality at three months.

Individual patient-data meta-analysis of three single-center RCTs comparing GA versus procedural sedation in anterior circulation strokes showed that the GA group had a higher proportion of mRS scores of 0–2 at 3 months, likely due to higher rates of mTICI scores of 2b or 3 (85.2% vs. 75.7% in the procedural sedation group) [10]. However, the only multicenter RCT to date (AMETIS trial)—including 273 patients with anterior circulation acute ischemic stroke—showed neutral results. Regarding posterior circulation stroke, the only RCT study in this subgroup of patients performed in two centers in China also provided neutral clinical results but with procedural superiority of GA group (mTICI 2b–3 of 95.3% vs 77.3% in sedation group). Our non-randomized observation of higher mortality in GA-treated posterior circulation strokes may provide additional perspective on patients with refractory occlusions and those requiring intracranial stenting, a likely underrepresented population in trials for which evidence remains limited. At present, any causal relationship between GA, clinical outcomes and mortality in thrombectomy patients remains unproven and in need of further randomized data.

We found a similar rate of procedural complications in both GA and local anesthesia/conscious sedation groups. Only intraprocedural contrast extravasation/vessel perforation was significantly higher in the local anesthesia/conscious sedation group. In the AMETIS multicenter RCT, the rate of arterial dissection or perforation was similar in GA and procedural sedation groups. Interestingly, 38.4% of patients in procedural sedation were agitated at some point during EVT. A pooled analysis of three single-center RCTs reported low rates of ICH/SAH in both groups: 5/185 (4.1%) cases in procedural sedation vs. 1/183 (0.8%) in the GA group (*p* not tested due to low numbers). Observational data from a large cohort of 4429 patients prospectively enrolled in the Italian Registry of Endovascular Treatment in Acute Stroke showed no significant difference in SAH between the GA (3.8%), conscious sedation (2.0%), and local anesthesia (2.6%) groups. Other studies reported similar findings, even in distal occlusions.

### Limitations

Our study has several limitations. It was a retrospective observational study with unmeasured confounders. The choice of anesthetic strategy was nonrandomized and subject to inherent biases, likely influenced by clinical factors; as such, general anesthesia was probably offered to patients with more critical presentations. The multicenter nature of the registry, while providing worldwide real-world data, also implies potential heterogeneity in anesthesia practices and peri-procedural management across sites. In fact, there was marked variability in anesthesia practice across centers. Most institutions predominantly used either sedation/local anesthesia or general anesthesia, rather than a balanced distribution of both modalities. We did not collect data on specific anesthetic drugs or types of GA (intravenous and/or volatile agents) or specific sedation strategies (local anesthesia, monitored anesthesia care and/or conscious sedation). Additionally, blood pressure measurements were not recorded, limiting our ability to assess the hemodynamic impact of the anesthetic strategy. The rate of conversion to GA was also not documented and patients in the GA group may have been intubated and extubated at different timepoints which may have influenced results. Potentially relevant clinical data, such as the incidence of pneumonia and intubation-related complications, were not recorded. Furthermore, we did not collect data on patient movement to assess the efficacy of conscious sedation.

Limitations aside, this study provides large, multicenter real-world evidence on the associations between two distinct anesthesia modalities and outcomes in patients submitted to intracranial stenting during endovascular stroke treatment.

## Conclusion

General anesthesia was not associated with improved technical or functional outcomes in patients who underwent acute intracranial stenting during endovascular stroke treatment. In-hospital and 90-day mortality was higher in patients who received general anesthesia, especially in posterior circulation strokes, likely reflecting an indication bias. Prospective, randomized studies are warranted to properly assess this effect.

## Supplementary Information

ESM1: Supplementary material 1
