# Supplementary material for: Anesthesia Modality in Intracranial Stenting for Acute Stroke—A Sub-Analysis of the RESISTANT International Registry
Source: Clin Neuroradiol. 2026 Feb 3;36(2):635–45. doi: 10.1007/s00062-026-01619-7 (PMC13319975; doi:10.1007/s00062-026-01619-7)

Supplementary table 1. Distribution of Anesthesia Type by Center

	Sedation/Local, n (%)	General Anesthesia, n (%)	Total, n
Center 1	0 (0.0%)	64 (100.0%)	64
Center 2	46 (92.0%)	4 (8.0%)	50
Center 3	31 (63.3%)	18 (36.7%)	49
Center 4	15 (32.6%)	31 (67.4%)	46
Center 5	31 (73.8%)	11 (26.2%)	42
Center 6	19 (46.3%)	22 (53.7%)	41
Center 7	28 (70.0%)	12 (30.0%)	40
Center 8	27 (87.1%)	4 (12.9%)	31
Center 9	0 (0.0%)	31 (100.0%)	31
Center 10	0 (0.0%)	31 (100.0%)	31
Center 11	14 (48.3%)	15 (51.7%)	29
Center 12	15 (53.6%)	13 (46.4%)	28
Center 13	13 (48.1%)	14 (51.9%)	27
Center 14	22 (84.6%)	4 (15.4%)	26
Center 15	13 (50.0%)	13 (50.0%)	26
Center 16	10 (38.5%)	16 (61.5%)	26
Center 17	12 (50.0%)	12 (50.0%)	24
Center 18	6 (26.1%)	17 (73.9%)	23
Center 19	17 (77.3%)	5 (22.7%)	22
Center 20	1 (4.5%)	21 (95.5%)	22
Center 21	18 (90.0%)	2 (10.0%)	20
Center 22	12 (63.2%)	7 (36.8%)	19
Center 23	14 (73.7%)	5 (26.3%)	19
Center 24	16 (94.1%)	1 (5.9%)	17
Center 25	12 (75.0%)	4 (25.0%)	16
Center 26	0 (0.0%)	14 (100.0%)	14
Center 27	11 (78.6%)	3 (21.4%)	14
Center 28	10 (76.9%)	3 (23.1%)	13
Center 29	0 (0.0%)	12 (100.0%)	12
Center 30	5 (41.7%)	7 (58.3%)	12
Center 31	0 (0.0%)	10 (100.0%)	10
Center 32	5 (55.6%)	4 (44.4%)	9
Center 33	6 (66.7%)	3 (33.3%)	9
Center 34	0 (0.0%)	8 (100.0%)	8
Center 35	2 (33.3%)	4 (66.7%)	6

Data are presented as number (row percentage). Percentages are calculated within each center.

Supplementary Figure 1. Distribution of 90-day mRS stratified by anterior and posterior circulation strokes

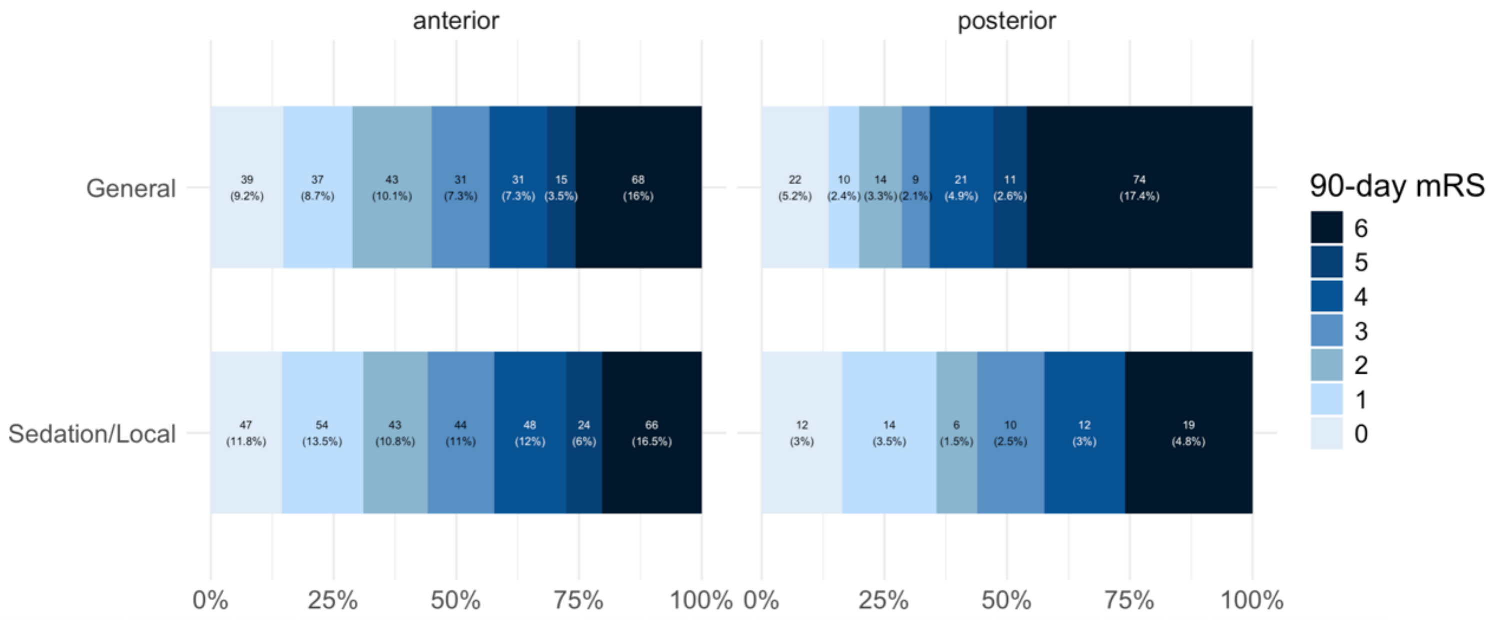

Supplement: Supplementary file 1 — ESM1: Supplementary material 1 [file 62_2026_1619_MOESM1_ESM.pdf]
